# Lifestyle Parameters in Patients with Diabetes Mellitus and in the General Adult Population—Trends over Five Years: Results of the Austrian National Health Interview Series

**DOI:** 10.3390/ijerph18189910

**Published:** 2021-09-21

**Authors:** Thomas Ernst Dorner, Christian Lackinger, Sandra Haider, Katharina Viktoria Stein

**Affiliations:** 1 Social Insurance Fund for Public Service, Railway and Mining Industries, 1080 Vienna, Austria; thomas.dorner@bvaeb.at (T.E.D.); christian.lackinger@bvaeb.at (C.L.); 2 Karl-Landsteiner Institute for Health Promotion Research, 3454 Sitzenberg-Reidling, Austria; 3 Department of Social and Preventive Medicine, Center for Public Health, Medical University of Vienna, 1090 Vienna, Austria; sandra.a.haider@meduniwien.ac.at

**Keywords:** diabetogenic, health behaviour, exercise, nutrition, smoking

## Abstract

Background: Not smoking, performing >150 min of aerobic physical activity (PA) and muscle strengthening exercises/week, and consuming >5 portions of fruit and vegetables/day are lifestyle recommendations for both the general population and people with diabetes mellitus (DM). Methods: A total of 15,771 and 15,461 persons from the Austrian Health Interview Surveys 2014 and 2019, respectively, including 4.9% and 6.0% of people with DM, were analysed in terms of their smoking, PA, and nutritional behaviours. Logistic regression models were performed for the lifestyle factors, adjusted for socio-demographic and health-related factors. Adjusted interactions between the survey year and DM on the lifestyle factors were computed. Results: The proportions of smokers were 23.9% and 20.2%, of people complying with the PA recommendations were 24.9% and 21.4%, and with fruit and vegetables recommendations were 7.1% and 5.5%, respectively, with significantly lower proportions of smokers and persons complying with the PA recommendations among people with DM. The fully adjusted odds ratios (95% confidence interval) for people with DM were 1.09 (0.94–1.26), 1.44 (1.23–1.69), and 0.90 (0.71–1.13) for smoking, not complying with PA recommendations, and not complying with fruit and vegetables recommendations, respectively. The proportion of people complying with PA recommendations decreased to a greater extent (*p* < 0.001) in people with DM (16.5% to 8.3%) compared to people without DM (25.3% to 22.3%). Conclusion: Diabetogenic lifestyle behaviours increased in the general Austrian population in recent years, which was especially true for people with DM regarding PA.

## 1. Introduction

Lifestyle measures are key interventions in the management of diabetes mellitus (DM), both to control the disease and to mitigate the risk of progression or exacerbation [1,2]. There is an abundance of literature and evidence available detailing the different recommendations for nutrition, physical activity (PA), stress reduction, alcohol intake, and smoking cessation for people with DM [3,4,5,6]. These lifestyle measures are also recommended for primary prevention of DM, as well as in pre-diabetes [4,6]. In order to be effective, these lifestyle changes need to be sustained over the life course, and each additional measure increases the beneficial and protective effects [2,7]. Adherence to such lifestyle recommendations, however, often requires improvement, both in the general population as well as in people with chronic conditions such as DM [8,9,10,11,12].

Additionally, circumstances change over time, as do lifestyle factors. Unforeseen, traumatic or positive events have additional impacts on lifestyle choices, such as eating habits, thereby influencing the adherence to recommendations [13]. Additionally, public health measures and policies to promote healthy lifestyles, such as anti-smoking policies [12,14,15], nutritional regulations [16,17,18], or policies to promote PA [19,20], have impacts on the health behaviour of people with and without DM. While the overall health outcomes still point towards increases in unhealthy weight, sedentary lifestyles, and unhealthy eating habits, these public health policies do show that health behaviours of populations can also change for the better and that they can be influenced by policy and practice. It is likewise well established that such lifestyle changes are difficult to maintain and that there are many different influencing factors determining the health behaviour of individuals [21,22]. These also include socio-demographic factors such as age, sex, educational level, comorbidities, and living conditions [10,21].

It was, therefore, the aim of this study to analyse these indicators of health behaviour in patients with DM in comparison to the general population and to observe whether these health behaviours changed over time. Furthermore, the study explored whether the changes over time occurred in equal measures in people with DM as in persons without the disease. These results will help to inform whether changes in health promotion and public health programmes need to be considered in the prevention and management of DM.

## 2. Material and Methods

### 2.1. Datasets

The Austrian Health Interview Surveys (ATHIS) for the years 2014 [23,24] and 2019 [25] were used for the analyses. This series of surveys represents the Austrian version of the European Health Interview Survey, which is mandatory for EU member states [26]. Both surveys were carried out by the Austrian National Statistics Agency, Statistik Austria, in the general population aged 15 years and older. The basis for the sample was the national central population register in 32 geographical regions. For most regions, the same number of participants was included, with a higher number for the three regions encompassing the Austrian capital, Vienna. Missing values were imputed after fundamental analysis of the non-responders based on sex, age, education, and region of residence. 

The ATHIS 2014 was carried out via computer-assisted telephone interviewing (CATI) between October 2013 and June 2015. The cross-sample size drawn from the national central population register was 38,768 subjects. Out of this, the data for 15,771 subjects were eligible for inclusion in the analysis, representing a response rate of 40.7%. In addition to the CATI questions, paper questionnaires were sent out to the respondents for specific topics, including the questions about PA, which had to be returned through regular post. The response rate for this part of the survey was 93% [24].

The ATHIS 2019 was carried out via computer-assisted personal interviewing (CAPI) and a web-based questionnaire between October 2018 and September 2019. The cross-sample size drawn from the national central population register was 30,608 subjects. Out of this, the data for 15,461 subjects were eligible for inclusion in the analysis (14,225 subjects were reached with CAPI and 1236 persons filled in the web-based questionnaire), representing a response rate of 50.5%. Similar to ATHIS 2014, ATHIS 2019 also included a self-administered questionnaire for specific topics, including the questions about PA. This self-administered part was carried out by the participants on an electronic device of their choice (e.g., laptop, desktop, iPad). Only a minority (1.5%) of the participants preferred a paper–pencil version of the self-administered questionnaire, the answers to which were digitalised upon receipt [25]. This study was guided by the STROBE checklist for epidemiological studies [27].

### 2.2. Variables

Smoking status: Daily cigarette smoking was indicated in the ATHIS 2014, if subjects answered “Yes, daily” to the question “Do you smoke?” and answered with “Cigarettes” to the question “Which of the following tobacco products do you use most frequently?”. In the ATHIS 2019, daily cigarette smoking was indicated, if the subjects answered “Yes” to the question “Do you smoke cigarettes daily?”. 

Physical activity: In evaluating the amount of PA, the 8-item PA Questionnaire of the EHIS (EHIS-PAQ) was used [28]. Compliance with the aerobe recommendations [29] was calculated by summing up the minutes per week spent cycling, playing sports, or engaged in fitness or recreational (leisure) physical activities. As international and national guidelines recommend at least 150 min moderate intensity PA per week [29], the indicator was dichotomised as fulfilling this criterion or not. Similarly, complying with the muscle-strengthening PA recommendations was indicated when the respondents answered that they performed muscle-strengthening activities (such as strength training or strengthening exercises with weights, with gymnastic bands, or with their own body weight, such as squats, push-ups, and sit-ups) at least twice a week. This is also in line with international and national recommendations and was also dichotomised as fulfilling this criterion or not. Finally, complying with both components of the PA recommendations was indicated if both aerobic and muscle-strengthening PA was performed according to the guidelines. 

Nutrition: Regarding nutrition, the respondents were asked how often per week they were eating fruits and vegetables, respectively, and if they answered with “Daily or more times per day”, they were asked to indicate, how many portions of fruits and vegetables, respectively, they were eating per day. The respondents were told that one portion was approximately a handful of fruit or vegetable, as a general rule. Since guidelines recommend the consumption of ≥5 portions of fruit or vegetables per day, with an emphasis on vegetable consumption [30], we calculated (1) the proportion of people who ate ≥2 portions of fruit, (2) the proportion of people who ate ≥3 portions of vegetables, and (3) the proportion of people who ate ≥5 portions of fruit or vegetables per day. This is in line with the Austrian nutritional guidelines [31] and the latest review of the evidence [30].

Diabetes mellitus: Regarding DM, subjects were asked whether they had DM as a diagnosed chronic disease in the past 12 months. No distinction between the different types of DM was made, as this is not mandatory in the EHIS and the ATHIS follows the EHIS format.

Socio-demographic and health-related variables: Participants’ sex (ATHIS only distinguishes between male and female) and age were recorded. Age was used in three categories (15–29 years, 30–64 years, and ≥65 years). The relationship status was represented as either ‘in a relationship’ (including being married) or ‘not in a relationship’. Education was defined in three levels: primary education (compulsory education up to age 15); secondary education (apprenticeship and vocational schools, professional or commercial schools, and high school); tertiary education (university). Furthermore, country of birth was documented into three categories: Austria; EU or EFTA states (comprising the 27 European member states for the years 2014 and 2019, plus the 4 EFTA states, except Austria); non-EU and non-EFTA states. The presence of a chronic disease was assessed with the question “Do you have a chronic disease or a chronic health problem?”. Finally, the body mass index (BMI) was calculated with the reported data for body weight and body height and categorised as underweight (<18.5 kg/m^2^), normal weight (18.5–24.9 kg/m^2^), overweight (25–29.9 kg/m^2^), or obese (≥30 kg/m^2^).

### 2.3. Statistics

All analyses were carried out using IBM SPSS Statistics 22 (IBM Corp., Armonk, NY, USA). The sample was weighted using the geographical region, age in 5 year groups, sex, family status, migration background, and educational level, as the weighting factors. Bivariate analyses were performed by means of cross-tabulations and group differences were assessed with Pearson’s Chi-squared tests.

Before including all variables into the multivariate model, the correlation of the variables with each other was tested by applying Pearson’s correlation coefficient in order to avoid problems of multicollinearity. The variables with the highest correlation were ‘presence of DM’ and ‘presence of any chronic diseases’, with a correlation coefficient of 0.236. There is no international standard for correct cut-off points for detecting multicollinearity [32], even though the most common threshold is 0.8 [33]. Thresholds of 0.5 and above have also been reported in the literature [34]. Based on this literature, we took 0.8 as the threshold. Since the highest value in our analyses was 0.236, all variables were included.

In order to test for the interaction between the year of evaluation and the presence of DM on the likelihood of the respective health behaviours, binary logistic regression analyses were carried out. In these analyses, the dependent variables were the different health behaviours, and we adjusted for the year of the survey, the presence of DM, and all socio-demographic and health-related variables. The *p*-value for the interaction between the year and the presence of DM is presented. Finally, we calculated binary logistic regression analyses with the respective health behaviour as the dependent variable, and all socio-demographic and health-related variables as the independent variables. The estimates of the logistic regression models with all mutually adjusted socio-demographic and health-related variables on the likelihood of having an unfavourable health behaviour are presented as ORs and 95%  CIs.

## 3. Results

The characteristics of the samples from both surveys are depicted in Table 1. Compared to the sample in 2014, the participants in 2019 were significantly more represented in older age groups, in categories with higher education, were more often gainfully employed, were more often born in a country outside Europe (non-EU or non-EFTA), were more often single, and were more often affected by a chronic disease. The prevalence of self-reported DM increased from 4.9% to 6.0%. Additionally, the prevalence rates of overweight and obesity rose significantly to 34.5% and 16.6%, respectively. On a positive note, the prevalence of daily smoking decreased significantly; however, the proportion of persons not complying with the two components of the PA recommendations (each and combined), as well as the proportion not complying with the recommended amount of daily fruit or vegetable consumption, fell significantly as well. Not even half of the Austrian population fulfilled the aerobe recommendations, less than one-third reached the muscle-strengthening recommendations, and just one-fifth managed to perform according to both PA groups. Similarly, just over one-quarter of the Austrian population ate 2 portions of fruit a day, while the proportions of people eating three portions of vegetables or 5 portions of fruit and vegetables were negligible at just below (4.3%) and above (5.5%) the benchmark, respectively.

People with DM smoked significantly less often compared with persons without DM. The proportions of daily smokers decreased from 2014 to 2019, both in patients with DM and in those without the disease. Additionally, people with DM were even less likely to meet the PA recommendations, which also was a significant result. The proportion of people complying with the recommendations decreased from 2014 to 2019; however, according to the significant interaction, differences were observed between patients with DM and people without DM. Regarding compliance with the aerobic and total PA recommendations, a stronger decrease was observed in patients with DM compared to persons without DM. Furthermore, there were also significant differences in the proportions of people with DM and those without DM who ate at least two portions of fruit per day (in both surveys) and who ate at least 3 portions of vegetables per day (in the survey 2014). The proportions of subjects who ate the recommended amount of fruit or vegetables decreased from 2014 to 2019, both in persons with and without DM (Table 2).

As shown in Table 3, there was no significant association between DM and the likelihood of daily smoking in the fully adjusted model. Factors associated with daily smoking were male sex, being in the middle age group, lower education, unemployment, being born abroad, living in the capital, not being in a relationship, and being underweight. There was a significantly lower likelihood for daily cigarette smoking in the 2019 survey compared to 2014 in the fully adjusted model. 

Table 4 shows that having DM showed a significant association with non-compliance with the PA recommendations (aerobic, muscle strengthening, and both combined) in the fully adjusted models. Additional factors associated with not complying with the PA recommendations were female sex, being in the higher age groups, having a lower education, being born abroad, being in a relationship, having a chronic disease, and not having normal weight. Additional factors for not complying with the aerobe component of the PA recommendations were unemployment and living in the capital (whereas living in the capital was associated with a higher likelihood of complying with the muscle-strengthening recommendations). In the fully adjusted model, there was a significantly higher likelihood for not complying with the PA recommendations in the 2019 survey compared to 2014.

As shown in Table 5, DM was significantly associated with a lower likelihood of not eating the daily amount of fruit in the fully adjusted model, whereas there was no significant association with the recommended amount of vegetable consumption or fruit and vegetable consumption. Male sex, lower education, and being born abroad were associated with a lower likelihood of eating fruit and vegetables in the recommended amounts. People in higher age groups ate fruit significantly more often but ate vegetables less often, and less often complied with the combined fruit and vegetable recommendations. There was a significantly higher likelihood for not complying with the recommended amount of fruit and vegetables in the 2019 survey compared with 2014 in the fully adjusted model.

## 4. Discussion

### 4.1. Unhealthy Behaviours Continue to Grow despite National and International Campaigns 

The overall international trends toward unhealthy weight, little PA, and insufficient fruit and vegetable intake are mirrored in this Austrian cohort, and the situation has clearly deteriorated over time. These developments in themselves are already cause for much concern, as higher weight, a lack of PA [35], and unhealthy nutritional habits [36,37] are all considerable risk factors for DM [1,38,39]. Indeed, our data showed that in parallel with the lifestyle changes in the general population, the prevalence of DM has increased. When taken together with alcohol intake and smoking, the mentioned lifestyle factors may account for between 75% and 91% of preventable cases of type 2 DM [2,37], apart from being associated with higher mortality rates in persons with DM [7], as well as in the overall population, even though the directionality seems to be similar for both [40]. Considering that only around one-quarter of Austrians without DM comply with the PA recommendations and eat at least 2 portions of fruit per day, it is clear that more than just public health measures and awareness campaigns are needed to change the lifestyle habits of the general population and to create awareness of the considerable risks these lifestyle choices carry. Even though discussions and information campaigns on these various risk factors and on healthy lifestyle choices have also been around for many years, they do not seem to have any effect on the population at large. In order to prevent DM successfully, all health promotive activities need to be taken together, and campaigns must also be designed considering environmental aspects such as availability and affordability of fresh produce, street safety, and workplace restrictions [39] in order to foster healthy living, working, and leisure environments that promote PA and healthy choices, not only in the form of sports, but also in terms of active commuting and providing affordable local fresh produce markets. This means that rather than focussing on the disease, new policies and programmes need to focus on enabling people to lead healthier lives, using health promotion as the guiding principle rather than disease prevention [2,7,35,38,39].

### 4.2. The Role of Social Determinants of Health

Social determinants of health are another important factor in tackling lifestyle changes. Austrian data follow other countries in that women eat healthier than men but are less physically active, and that migrants from outside the EU tend to have worse health outcomes than people born in Austria or the EU. Investments in multi-faceted and targeted programmes covering all settings pay dividends, as they considerably contribute to the prevention of DM and reduce mortality risks in people with DM, as mentioned above [2,7]. Moreover, in high-risk patients (e.g., people with overweight, sedentary behaviour, or smokers), lifestyle interventions including weight management, PA, and nutrition reduce the risk of developing type 2 DM by 40–50% [41].

### 4.3. Self-Management and Care Coordination Are Crucial to Support Sustainable Lifestyle Changes

The analysis also highlights that adults with DM are even less compliant with the recommended lifestyle adjustments than the general population. As non-compliance with recommended lifestyle choices can lead to the progression of DM, the development of co-morbidities, or preventable exacerbations [1,39,41], this result gives rise to even more concern. Disease management programmes for DM have tried to address this issue, with varying and ambivalent success [42,43]. Some of the reasons named for the limited outcomes have been a lack of co-designing measures with patients and their families, a misguided focus on DM while ignoring other health and care needs, and short interventions with no evaluation and follow-up [43]. On the positive side, self-management support [44] and multi-disciplinary care coordination have continuously shown impacts through better health outcomes in patients with DM [43]. Overall, it seems clear that even with the manifold health literacy programmes available for people with DM, the information does not reach them or does not help them to make sustainable lifestyle choices. It is, therefore, necessary for the health system, especially primary care providers and public health organisations, to expand their traditional approaches to DM management and think about more engaging ways of involving people with DM actively in their own care [45,46].

### 4.4. The Need for Multi-Faceted and Enabling Health Management Programmes

The data and development over time in conjunction with the international evidence show that in order to reverse these trends, renewed efforts need to concentrate on bringing everything together in multi-faceted programmes that address all aspects of a healthy lifestyle, taking into account the necessity of easy applicability in everyday life and cutting across different settings. These approaches need to coordinate and align efforts from public health agencies with those in primary care and short-term inpatient programmes. They should include comprehensive approaches that take into account family and workplace situations and encourage not only obvious recommendations around PA and nutrition, but that also look at mental health, functional and critical healthy literacy (i.e., the ability to act upon information received) [47], and social capital [48]. 

A good example is the Finnish implementation programme for the prevention of type 2 DM (FIN-D2D), which combines integrated care guidelines with national incentives such as taxation to promote healthy food choices, short-term intervention programmes, and a comprehensive follow-up and support system in primary care [49]. In social insurance systems, social security institutions are well placed to take a leading role, as they collaborate with primary care provides, provide evidence-based health information for those they insure, and offer short-term programmes in dedicated facilities. 

### 4.5. Strengths and Limitations

The strengths of the study include the large and representative sample sizes of the two surveys and the fact that the Austrian Central Population Register was used to draw the samples, which represents the entire Austrian population. In contrast to studies from clinical settings, the population-based design of our study permitted the combined analysis of healthy subjects with people with DM. The limitations of the study include the slightly different methods used (CATI in 2014 vs. CAPI in 2019), which could have yielded an underestimation of unfavourable health behaviour in 2014, since unfavourable behaviour is usually reported more honestly and more often in personal interviews compared to telephone interviews [50,51]. Additional limitations are the self-reported nature of all variables, which may have led to underreporting of diseases such as DM and of socially undesirable lifestyle habits, as well as the fact that we could not distinguish between type 1 and type 2 DM. Furthermore, it has to be mentioned that the analysed surveys were cross-sectional datasets; therefore, temporal and causal conclusions regarding the associations between lifestyle factors and DM cannot be drawn from our findings.

## 5. Conclusions

People with DM show less favourable lifestyle behaviours compared to the general population, although unhealthy lifestyle behaviours have increased overall. This illustrates again that even though the prevention and management of DM has been widely studied over the past decades, with numerous guidelines and disease management programmes existing, long-term success remains elusive and limited. The reasons are manifold, although it is clear that well-defined measures and recommendations by themselves are not enough to enable people to sustainably change their lifestyles. As with all health promotion and prevention measures, it is necessary to co-design care plans together with the population and to support them over their life course, not only through clinical interventions, but more importantly by providing healthy environments that promote healthy choices.

## Figures and Tables

**Table 1:** Socio-demographic and health-related characteristics of participants in the Austrian Health Interview surveys 2014 and 2019 (all values in %).

	2014	2019	*p* Value *
Male	48.6	48.9	0.704
Age			0.004
15–29	21.5	20.1	
30–64	57.5	57.8	
65+	21.0	22.0	
Education level			<0.001
Primary	22.3	19.5	
Secondary	64.3	64.1	
Tertiary	13.4	16.4	
Employment status			0.001
Gainfully employed	52.3	53.8	
Unemployed	5.1	4.4	
Not gainfully employed	42.6	41.8	
Country of birth			<0.001
Austria	82.9	80.4	
EU/EFTA	10.7	9.1	
Non-EU/Non-EFTA	6.5	10.5	
Status of living			
Living in Vienna	20.8	21.2	0.446
In a relationship	65.4	60.5	<0.001
Body Mass Index			<0.001
Underweight	2.8	2.5	
Normal weight	50.5	46.3	
Overweight	32.4	34.5	
Obese	14.3	16.6	
Lifestyle variables			
At least one chronic disease (yes)	36.0	38.3	<0.001
Diabetes mellitus (yes)	4.9	6.0	<0.001
Daily cigarette smoking (yes)	23.9	20.2	<0.001
Physical activity			
Complying with the aerobe recommendations	50.1	43.2	<0.001
Complying with muscle strengthening recommendations	33.3	28.9	<0.001
Complying with both components	24.9	21.4	<0.001
Nutrition			
≥2 portions fruit daily	29.7	26.4	<0.001
≥3 portions vegetables daily	5.0	4.3	0.002
≥5 portions fruit or vegetables daily	7.1	5.5	<0.001

Note: * *p* values as results of the Chi^2^ test between 2014 and 2019.

**Table 2:** Health behaviour in people with and without diabetes mellitus in the Austrian Health Interview surveys 2014 and 2019 (all values in %).

	2014			2019			Interaction between Year and DM on Respective Lifestyle Factor
	People with DM	People without DM	*p* Value *	People with DM	People without DM	*p* Value *	*p* Value **
	**Smoking**						
Prevalence of daily cigarette smoking	17.1	24.3	<0.001	17.4	20.4	0.027	0.093
	**Physical activity**						
Compliance with the aerobe recommendations	37.1	50.7	<0.001	21.8	44.6	<0.001	<0.001
Compliance with muscle-strengthening recommendations	25.2	33.7	<0.001	17.7	29.6	<0.001	0.066
Compliance with both components	16.5	25.3	<0.001	8.3	22.3	<0.001	<0.001
	**Nutrition**						
≥2 portions fruit daily	34.9	29.4	0.001	30.5	26.1	0.003	0.616
≥3 portions vegetables daily	3.2	5.1	0.019	3.1	4.3	0.071	0.480
≥5 portions fruit or vegetables daily	6.6	7.1	0.558	4.2	5.6	0.070	0.351

DM = diabetes mellitus. Note: * *p* values as results of the Chi^2^ test between persons with and without DM; ** *p* values for the interaction between year and presence of DM in binary logistic regression analyses with the respective health behaviour as the dependent variable, adjusted for year of the survey, the presence of DM, and all socio-demographic and health-related variables.

**Table 3:** Associations of diabetes mellitus, socio-demographic and health-related factors, and year of survey with the likelihood of daily cigarette smoking in the Austrian Health Interview surveys 2014 and 2019.

	OR	95% CI
Diabetes mellitus No diabetes mellitus	1.09 1	0.94–1.26
Male Female	1 0.83	0.78–0.87
15–29 30–64 65+	1 1.14 0.36	1.06–1.23 0.32–0.41
Primary Secondary Tertiary	3.50 2.91 1	3.12–3.93 2.64–3.21
Gainfully employed Unemployed Not gainfully employed	1 1.82 0.54	1.63–2.04 0.50–0.58
Austria EU/EFTA Non-EU/Non-EFTA	1 1.50 1.25	1.37–1.64 1.13–1.37
Vienna Other federal states	1.44 1	1.35–1.55
In a relationship Not in a relationship	1 1.37	1.29–1.46
At least one chronic disease No chronic disease	1.12 1	1.05–1.19
Underweight Normal weight Overweight Obese	1.36 1 0.98 0.93	1.15–1.60 0.92–1.05 0.85–1.01
2014 2019	1 0.81	0.76–0.85
R^2^	0.133	

**Table 4:** Associations of diabetes mellitus, socio-demographic and health-related factors, and year of survey with the likelihood of not complying with the components of the physical activity recommendations in the Austrian Health Interview surveys 2014 and 2019.

	Not Complying with the Aerobe PA Recommendations		Not Complying with Recommendations for Muscle Strengthening PA		Not Complying with Both Components of PA Recommendations	
	OR	95% CI	OR	95% CI	OR	95% CI
Diabetes mellitus No diabetes mellitus	1.38 1	1.23–1.54	1.27 1	1.12–1.44	1.44 1	1.23–1.69
Male Female	1 1.29	1.32–1.36	1 1.39	1.32–1.46	1 1.52	1.44–1.61
15–29 30–64 65+	1 1.29 1.82	1.21–1.38 1.68–1.98	1 1.57 1.68	1.47–1.68 1.54–1.84	1 1.58 1.96	1.47–1.70 1.78–2.16
Primary Secondary Tertiary	2.43 1.71 1	2.23–2.64 1.60–1.83	1.85 1.39 1	1.69–2.02 1.30–1.49	2.04 1.51 1	1.85–2.25 1.40–1.63
Gainfully employed Unemployed Not gainfully employed	1 1.18 0.84	1.05–1.33 0.79–0.89	1 1.06 0.89	0.93–1.19 0.84–0.95	1 1.11 0.87	0.97–1.27 0.81–0.94
Austria EU/EFTA Non-EU/Non-EFTA	1 1.21 1.78	1.12–1.31 1.62–1.95	1 1.08 1.34	1.00–1.18 1.21–1.47	1 1.14 1.36	1.04–1.25 1.22–1.52
Vienna Other federal states	1.32 1	1.25–1.40	0.93 1	0.88–0.99	1.06 1	0.99–1.14
In a relationship Not in a relationship	1 0.92	0.87–0.97	1 0.79	0.75–0.83	1 0.80	0.75–0.85
At least one chronic disease No chronic disease	1.32 1	1.25–1.39	1.09 1	1.03–1.15	1.20 1	1.13–1.28
Underweight Normal weight Overweight Obese	1.35 1 1.30 1.83	1.17–1.56 1.23–1.37 1.71–1.97	1.25 1 1.28 1.64	1.08–1.46 1.20–1.35 1.51–1.77	1.31 1 1.32 1.95	1.11–1.55 1.24–1.41 1.78–2.15
2014 2019	1 1.31	1.25–1.37	1 1.23	1.17–1.30	1 1.21	1.15–1.28
R^2^	0.091		0.057		0.072	

PA = physical activity.

**Table 5:** Associations of diabetes mellitus, socio-demographic and health-related factors, and year of survey with the likelihood of not consuming the recommended daily amount of fruit and vegetable in the Austrian Health Interview surveys 2014 and 2019.

	Not Consuming ≥ 2 Portions of Fruit/Day		Not Consuming ≥ 3 Portions of Vegetables/Day		Not Consuming ≥ 5 Portions of Fruit or Vegetables/Day	
	OR	95% CI	OR	95% CI	OR	95% CI
Diabetes mellitus No diabetes mellitus	0.84 1	0.75–0.94	0.93 1	0.63–1.24	0.90 1	0.71–1.13
Male Female	1 0.52	0.49–0.55	1 0.51	0.45–0.59	1 0.45	0.41–0.51
15–29 30–64 65+	1 0.84 0.71	0.78–0.91 0.65–0.78	1 1.42 3.17	1.24–1.63 2.58–3.88	1 1.28 1.81	1.13–1.45 1.53–2.12
Primary Secondary Tertiary	1.33 1.25 1	1.22–1.45 1.16–1.34	2.84 2.11 1	2.37–3.41 1.85–2.40	2.22 1.81 1	1.90–2.60 1.61–2.04
Gainfully employed Unemployed Not gainfully employed	1 1.19 0.80	1.04–1.35 0.74–0.85	1 1.20 0.72	0.90–1.60 0.63–0.81	1 1.25 0.82	0.98–1.61 0.73–0.92
Austria EU/EFTA Non-EU/Non-EFTA	1 0.75 0.67	0.69–0.82 0.61–0.73	1 0.79 0.82	0.68–0.93 0.68–0.98	1 0.61 0.63	0.53–0.70 0.54–0.74
Vienna Other federal states	1.06 1	0.99–1.13	0.80 1	0.71–0.91	1.02 1	0.91–1.14
In a relationship Not in a relationship	1 1.04	0.98–1.10	1 0.97	0.86–1.09	1 0.91	0.88–1.08
At least one chronic disease No chronic disease	1.09 1	1.03–1.15	0.97 1	0.86–1.10	0.97 1	0.88–1.08
Underweight Normal weight Overweight Obese	1.18 1 1.05 1.06	1.00–1.38 0.99–1.12 0.98–1.14	1.13 1 1.13 1.26	0.84–1.52 0.99–1.28 1.05–1.51	1.16 1 1.13 1.21	0.89–1.51 1.01–1.26 1.04–1.41
2014 2019	1 1.21	1.15–1.27	1 1.22	1.09–1.36	1 1.36	1.24–1.50
R^2^	0.051		0.059		0.053	

## Data Availability

The ATHIS survey data can be obtained from Statistik Austria for research and educational purposes. For more information, please visit: http://www.statistik.at/web_de/services/mikrodaten_fuer_forschung_und_lehre/datenangebot/standardisierte_datensaetze_sds/index.html (accessed on 2 August 2021).
